# Metal Additive Manufacturing of Plastic Injection Molds with Conformal Cooling Channels

**DOI:** 10.3390/polym14030424

**Published:** 2022-01-21

**Authors:** Baris Burak Kanbur, Yi Zhou, Suping Shen, Kim Hai Wong, Charles Chen, Abe Shocket, Fei Duan

**Affiliations:** 1 Singapore Centre for 3D Printing (SC3DP), Nanyang Technological University (NTU), Singapore 639798, Singapore; bbkanbur@ntu.edu.sg (B.B.K.); yi.zhou@ntu.edu.sg (Y.Z.); spshen@ntu.edu.sg (S.S.); 2 School of Mechanical and Aerospace Engineering, Nanyang Technological University (NTU), Singapore 639798, Singapore; 3 Tyco Electronics Singapore Pte Ltd., TE Connectivity, Singapore 239920, Singapore; kimhai.wong@te.com (K.H.W.); chen.charles@te.com (C.C.); ashocket@te.com (A.S.)

**Keywords:** metal additive manufacturing, 3D printing, computer-aided engineering, computer-aided design, conformal cooling, heat transfer, conjugate heat transfer, Direct Metal Laser Sintering, multiobjective optimization, plastic injection

## Abstract

Conformal cooling channels (CCCs) are widely used in the plastic injection molding process to improve the product quality and operational performance. Tooling that incorporates CCCs can be fabricated through metal additive manufacturing (MAM). The present work focuses on the MAM of a plastic injection mold insert with different CCC types that are circular, serpentine, and tapered channels with/without body-centered cubic (BCC) lattices. The entire manufacturing process of the mold insert is explained from the design step to the final printing step including the computational thermal & mechanical simulations, performance assessments, and multiobjective optimization. Compared to the traditional channels, conformal cooling channels achieved up to 62.9% better cooling performance with a better thermal uniformity on the mold surface. The optimum mold geometry is decided using the multiobjective optimization procedure according to the multiple objectives of cooling time, temperature non-uniformity, and pressure drop in the channel. Direct Metal Laser Sintering (DMLS) method is used for manufacturing the molds and the quality of the printed molds are analyzed with the X-ray Computed Tomography (X-ray CT) technique. The errors between the design and the printed parameters are less than 5% for the circular and tapered channels while the maximum deviation of the strut diameters of the BCC is 0.06 mm.

## 1. Introduction

Plastic injection is one of the most affordable and broad thermoplastic polymer-related processes for mass production purposes in the industry [1,2]. It has four main steps that are (in a sequence) (i) melt plastic injection, (ii) packing in the mold, (iii) cooling process and (iv) ejection from the mold [3]. The total consumed time during these steps is called total cyclic time whilst the cooling step shares 70% to 80% of the total cyclic time [4]. Notwithstanding that all these steps have crucial importance on the plastic injection molding process, insufficient or low-quality cooling can result in negative impacts on the product quality like unwanted warpage and shrinkage. In general, there are two critical parameters to manage the cooling process well: cooling time and temperature uniformity on the mold surface. The decrease of cooling time is the desired point for performance improvements of the cooling step of the plastic injection molds since it can decrease the risks of warpage and shrinkage while it also achieves shorter cyclic time, which allows greater production rates. However, if the cooling time decrement cannot be adjusted well, it results in an increment in shrinkage opposite of the target of shrinkage minimization. Therefore, the decrease of cooling time should be controlled with the temperature distribution on the mold surface that is called temperature uniformity. 

The cooling performance can be improved via two factors that are the mold material properties and the cooling application [5]. Between these two factors, the cooling application is preferable because the mold material properties are mostly decided according to the mechanical properties like strength, weight, etc. rather than considering the thermal conductivity value. Thus, the mold materials are made of stainless-steel types in most plastic injection molding applications and the mold material is not considered as the cooling performance improvement factor in practical applications unless otherwise indicated. Traditional mold cooling involves drilling straight channels in the mold geometry. They are easy-to-fabricate, economically feasible, but also very basic that can result in an insufficient cooling process, especially for the complex plastic models. The complexity of plastic products is defined according to the challenging curves of the product, which are very difficult to be followed by the straight pathways of the traditional channels. Hence, cooling time and temperature non-uniformity increase, and they cause low product quality with defective results. To improve the cooling performance, optimum cooling parameters like channel spacing (distance between two straight-drilled channels), channel diameter, flow rate, and thermal properties of the circulated coolant through the channel can be used [6,7]. Nevertheless, the mentioned improvement approaches can be still insufficient for the complex plastic products due to the design limitations of the channels and traditional machining.

Differently from the traditional machining tools, the recent developments in additive manufacturing (AM) technology make the fabrication of complex but effective channels in affordable ways. Hereby, AM can be effectively used in the plastic injection mold processes [8]. By means of the design and manufacturing flexibilities of AM tools [9,10], cooling channels can follow the complex contours of the plastic product in the mold geometry; hereby, they are named conformal cooling channels (CCCs). Compared to traditional channels, CCCs achieve a shorter cooling time that was found in the wide range of 15–50% with reference to refs. [11,12,13]. Beard [14] approximately calculated the profit income increment in the range of 27–55% corresponding to the decrement of cooling time by 20–40%. Dimla et al. [15] found that the product improvement rate can reach up to 70% when the CCCs are considered in the injection molding process. However, as mentioned above, temperature uniformity is also a crucial factor and these two factors (cooling time and temperature uniformity) do not always go in parallel with each other because uncontrolled short cooling time may result in temperature non-uniformity. Also, due to the complex channel pathways in the mold, the pressure drop values of the circulated coolant becomes higher than the obtained pressure drops in traditional channels, which cause extra pump consumption and decrease the energy efficiency of the plastic injection molding process. Thus, the design and optimization of CCCs are crucial steps before implementing in a real environment. 

Up to now, there have been various CCC applications for the plastic injection molding process. Different cross-sectional profiles are popular research motivations in the CCC development studies. Among different cross-sectional profiles, circular CCCs are generally the first-considered ones due to their simplicity. In ref. [16], the CCC design was presented with the cross-sectional profile alternatives of circular, trapezoid, and rectangular for liquid cooling purposes in a battery pack. Besides the profile type, number of CCCs, the diameter of CCCs, the distance between CCC and mold surface were investigated. The use of CCCs improved the cooling performance up to 39% while the product performance (shrinkage, warpage, etc.) was enhanced by nearly 5%. Saifullah and Masood [17] focused on the decrease of the total cyclic time of a plastic canister model in the plastic injection molding process. Comparing the traditional and conformal cooling channels, which both had circular cross-sectional profiles, it was seen that the CCCs reduced the totally cyclic time by up to 35% whilst the maximum temperature on the mold surface was decreased by nearly 30%. Following that study, Saifullah et al. [18] considered the same plastic model and improved the cooling time by 37.5% using the high-conductive copper-supported bi-metallic conformal cooling channels. Mazur et al. [19] performed a spiral pathway for the CCC design, which was not possible with traditional machining. The comparative study inferred that the spiral CCC provided 5 °C lower ejection temperature that is an improvement sign from the standpoints of shrinkage and warpage. Schmidt et al. [20] conducted the CCC solution in two different mold material types: P-20 steel and 420 SS/bronze. The use of CCCs improved the cooling time by 32.7 and 19.8% whilst the corresponding shrinkage was decreased by 28.2% and 15.1% for P-20 steel and 420 SS/bronze, respectively. For more detailed examples of CCC applications and comparative analyses between traditional channels and CCCs, previously published review works [3,4,21] can be referred to.

The aforementioned studies emphasize the importance of CCCs in the cooling step of the plastic injection mold process. It is also seen that each plastic product model has its own limitations so that the CCC design should be considered unique for each plastic product model. According to the product properties (e.g., polymer material properties, contours of plastic product-which affect the mold geometry-, injection & ejection temperature), CCCs may have different constraints. That is, the CCC application is a complex procedure from the initial design to the final printing process of the mold geometry. Even though the above-given literature and relevant review papers present useful insights, there are still research gaps for a better understanding of CCC applications. Therefore, we present a comprehensive procedure of the CCC fabrication from design to final product step using Direct Metal Laser Sintering (DMLS). For a specific mold geometry, we consider three different CCC types that are circular, elongated, and tapered CCCs. Also, using the flexibilities of the MAM technique, we integrated body-centered cubic (BCC) lattice structure into the tapered CCC and created another CCC type called BCC-integrated tapered CCC. After defining the design details, computational thermal simulations are explained with parametric performance maps. Then, the final dimensions of the CCCs are decided using the multi-criteria decision-making approach. Finally, the DMLS method is explained with printing parameters, and the final product (CCC-integrated mold geometry) is fabricated.

## 2. Outline of the Entire Process

**Figure 1:**
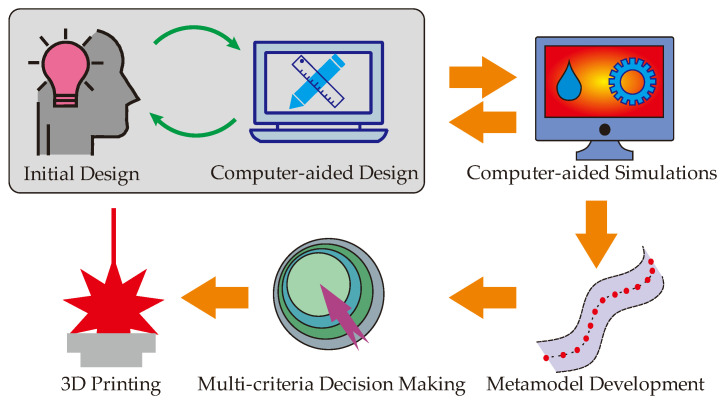
The entire process of the metal additive manufactured CCCs.

The entire process of the metal additive manufactured CCCs has six main steps from the initial design to the final fabrication. These main steps are common for all types of metal additive manufactured CCCs. However, additional steps or sub-steps can be integrated to this process; they depend on the selection & operation criteria of the designer, operator, and decision-maker. Figure 1 projects the main steps of the entire process for developing the metal additive manufactured CCCs.

The first step is building the design-related opinions. In this step, the MAM machine-related design limitations, metal powder-related constraints, thermal performance boundaries of the target plastic product, weight & size limitations of the mold body and CCCs, etc. are considered in detail. It is suggested to combine ideas from all parts of the product that are from designers, engineers, operators, and customers. Hereby, common ground on the desired mold design can be reached and the next step, computer-aided design (CAD), takes place. Using the benefits of a 3-dimensional (3D) design environment, both designers and operators are able to see detailed points of the initial design. Then, considering the printability (including the repeatability of the printed products), printing time, maintenance, operation, etc., the CCC-based mold geometry becomes ready for computer-aided simulations. As shown in Figure 1, a feedback mechanism (green arrows in the figure) should be built between the initial design and CAD steps.

Computer-aided simulations are the next step after the design-related steps, and it is strongly suggested for efficient analysis of the developed CCCs in the mold. Since the CCCs have complex pathways that follow the curves of the plastic product, their thermal & mechanical analyses are not as easy as the straight-drilled channels so that analytical thermal & mechanical models or the established correlations, which are feasible for straight-drilled channels in the manufacturing industry, are not applicable. To overcome these challenges, computer-aided simulations allow us a better understanding of thermal and mechanical performances before printing the product via MAM machines. Since the simulations are done in a computational environment, it is easy to re-design and re-simulate before finalizing the product design. Orange arrows in Figure 1 deduce the second feedback mechanism in the entire process. Once the desired thermal & mechanical performance data is obtained, the next step becomes the metamodel development. 

The metamodel development is a critical step between the computer-aided simulations and the final decision-making. Even though the computer-aided simulations allow us to analyze the thermal & mechanical performances of the mold geometry before printing it out, we still aim to reduce the computational process as much as possible because longer computational processes simply mean higher computational cost and more computational time. Therefore, we aim to minimize the computational process whilst we collect sufficient data for the understanding of the thermal & mechanical performances. That is, the performed simulations help to detect the lower and upper boundaries of the design space for the CCC-based mold geometry; they can also create additional simulation-based points in the design space. However, filling the design space with simulation data is computationally expensive and time-consuming so that the metamodeling approaches help up creating additional points in the design space. The metamodel definition is broad in engineering applications, in fact. However, for the CCC-related solutions, it can be considered as an approach of interpolation, which aims to calculate/predict accurate data among the simulated data in the design space. There are multiple types of metamodels for CCC-based mold geometries, and the selection of the metamodel should be done according to the dimensions of the design space. If the CCC-based mold geometry has three or fewer design variables, traditional interpolation approaches and neural networks are suggested to be applied. Both traditional interpolation approaches and neural network structures are efficient metamodels as they can process the calculation/prediction in a short time with accurate approximations. More details on traditional interpolation approaches and neural network applications can be found in refs. [22,23], respectively. In this study, we explain the entire process using a CCC-based mold geometry that has three different design variables so that the traditional interpolation or neural network structure is sufficient for accurate calculation/prediction. On the other hand, for the mold geometries that have a high number of design variables (4 or above), the traditional interpolation approaches are not able to provide accurate prediction since the dimension of the design space is large and results in significant uncertainties. Neural networks can still be applied for high dimensional design spaces, but it is also the fact that the accurate prediction via neural networks requires a large amount of input data for efficient training of the neural networks; this may result in too much computational effort. To deal with these challenges, a design of experiment (DoE) structure should be built before the computer-aided simulations. That is to say, a DoE structure, which can accurately work for high dimensional design spaces, should be located between the design and computer-aided simulation steps in Figure 1. Hereby, the number of computational simulations can be economically managed using the preferred simulation points by the DoE structure. When the DoE analysis is done and the computational simulations are completed, statistical or discrete computing approaches can be preferred as metamodeling tools instead of traditional interpolation approaches or neural network structures. In one of our recent works [24], we developed a hybrid method, called a combined Latin Hypercube Design (LHC) and Delaunay Triangulation (DT) approach. In this new method, the LHC structure (a type of DoE that is effective for high dimensional spaces) and the DT method are combined to accurately predict additional points in the high dimensional design space. Similar to this approach, other approaches can be developed for high dimensional cases. Independent of the number of design variables, the metamodel should provide a sufficient amount of data in the design space for the next step: multi-criteria decision making. 

The multi-criteria decision making is the final step before the printing operations. It has a wide definition and there are several ways (e.g., analytical, numerical, geometric, graphical, optimization, or combined approaches) for multi-criteria decision-making approaches. We prefer using multiobjective optimization for both low- and high-dimensional spaces. In general, multiobjective optimization considers multiple objectives of a specific engineering problem to present the best trade-off point in the defined upper and lower bounds. In this manuscript, three different objectives are defined for the plastic mold geometry; which are cooling time, temperature non-uniformity, and pressure drop. All these objectives are related to thermal performance in the current study; however, if requested by designers, customers, or engineers’ mechanical performance-related objectives like fatigue life or maximum stress can be considered as one of the multiple objectives. For example, Erturk et al. [25] experimentally investigated the mechanical performance of the lattice structures as microstructures. Nevertheless, the use of mechanical performance-related data (e.g., fatigue life) is recommended for constraints of the optimization problem instead of using them as objectives. For example, in the current study, all the developed design solutions must provide the mechanical strength of 1 million life cycles from the standpoint of fatigue life. The reason behind recommending mechanical performance as a constraint instead of one of the objectives is the basic logic of the manufacturing process. With the exceptions of some specific cases, the mechanical strength is generally asked for a minimum limit. If the mechanical strength is above the requested limit, the product is assumed satisfactory, and the manufacturing process continues as planned. This means that the minimum limit of mechanical performance-related data is vital while there are no strict conditions for the maximum limit. However, for thermal-fluid performance-related data like cooling time, temperature non-uniformity, and pressure drop; both minimum and maximum limits have pivotal roles so that they are suggested to be considered as objectives in the optimization problem. Once the design space is clarified with upper & lower bounds, the sufficient amount of data points via metamodels & computational simulations, and performance constraints; the multiobjective optimization procedure can be initiated. The multiple objectives of this study have minimization purposes since their minimum values mean shorter operation time, better product quality, and lower pump power requirement. However, in some cases, maximization can also be aimed. Therefore, minimization and maximization purposes should be defined very well before initiating the multiobjective optimization work. The results of the multiobjective optimization are plotted in the Pareto frontier screens so that the best trade-off point can be decided according to the weights of the objectives and/or preferences of the decision-maker, which can be human or machine. The selected best trade-off point represents the final design & optimization parameters of the desired CCC-based mold geometry. After this step, the final step is the MAM step. We use the Direct Metal Laser Sintering (DMLS) method in the current work; however, other techniques can be applied, inherently. The operating conditions of the printing process depend on the machine type and printing technique so that all the printing parameters should be assessed in detail to overcome potential faults or maloperations. In the sections below, each step is explained for a specific mold geometry. 

## 3. Design of CCC-Integrated Mold Geometries

### 3.1. Design of the Baseline (Straight-Drilled) Channel-Based Mold Geometry

**Figure 2:**
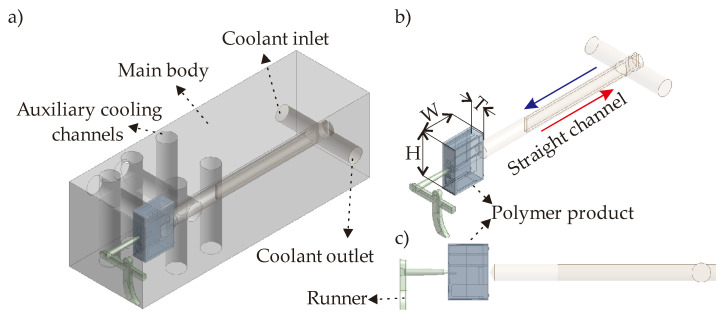
Design of the straight-drilled cooling channel: (**a**) isometric view of the whole body; (**b**) isometric view of the straight channel, polymer product, and runner; and (**c**) side view of the straight channel, polymer product and runner.

The initial design of the mold geometry should include the plastic part (polymer), runner, main & auxiliary cooling channels in addition to the aimed CCC design. Figure 2a illustrates the initial design of the mold geometry with a straight-drilled cooling channel including the plastic runner and main & auxiliary cooling channels. 

The presented design in Figure 2 is the baseline design of our CCC development concept. The baseline design is assumed with the cooling time of 7 s in the factory. The polymer product is polybutylene terephthalate (PBT) with the density of 1396.0 kg/m^3^, the specific heat of 1363 J/kgK, and thermal conductivity of 0.29 W/mK [26] whilst the mold body is steel with the density of 8030.0 kg/m^3^, the specific heat of 502.48 J/kgK, and thermal conductivity of 16.27 W/mK [22]. The dimensions of the polymer product are 14 × 20 × 6.5 mm (W × H × T in Figure 2b). In this study, the plastic injection temperature is defined as 543 K while the ejection step is activated at the temperature of 323 K. Reference cooling time and the plastic temperatures were determined from actual experiments performed apriori. This measurement data was used as reference data for validating the boundary conditions and results of the computational simulations. In Figure 2a, it is seen that the main body has also auxiliary cooling channels. The dimensions of these auxiliary cooling channels are not in the scope of this work as they have constant dimensions with respect to the real factory operations. The location and the number of channels do not change during the development of the CCC solutions. The proposed CCCs are considered to replace the straight channel given in Figure 2b,c. Here, a baffle was constructed that allows coolant flow-in in the direction of the blue arrow, and flow-out with the direction of the red arrow in Figure 2b. The water touches the mold insert of the target plastic product part at the end of the straight channel (Figure 2c). The main motivation behind developing CCCs for the polymer product in Figure 2 is proposing more effective channel pathways to better manage the temperature uniformity and the cooling time. However, longer CCC geometries result in higher pressure drops so that the main concept of this study is achieving. In order to achieve the best trade-off point for minimizing the cooling time, temperature non-uniformity, and pressure drop. For this purpose, five different CCC structures are proposed as (i) circular CCC, (ii) elliptical CCC, (iii) elongated CCC, (iv) triangular CCC, and (v) tapered CCC. 

### 3.2. Circular CCC-Integrated Mold Geometry

**Figure 3:**
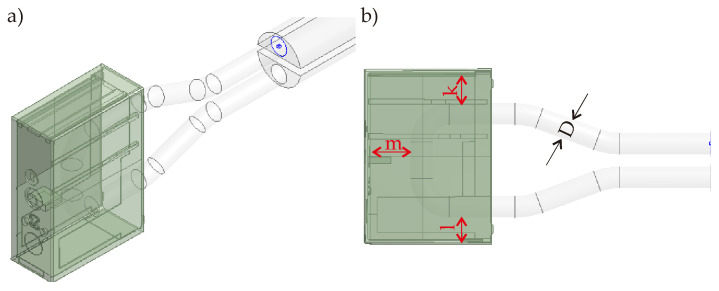
3D view of the circular CCC: (**a**) isometric view and (**b**) side view.

For the same polymer product, the first developed CCC solution is the circular CCC. The circular CCC integration is shown in Figure 3. Instead of point contact of the cooling channel like in traditional design (Figure 2), the MAM brings flexibilities during the design step so that the circular CCC has more complex channel curves, which come closer to the hot points of the polymer product and thereby provides better cooling time and temperature uniformity. 

The circular CCC has a constant diameter value, D, along the CCC pathway. The D value has a range of 2.1–2.5 mm. The reason behind selecting this range is directly related to the printability of the CCCs. The printability criteria depend on the machine type and the thermal boundary conditions of the melt polymer, which creates the hot spots and therefore should be managed well during the cooling process. More details on the printability concerns can be found in ref. [21]. The printability also affects the CCC pathway in the mold geometry. In the circular CCC, the CCC pathway is U-shaped (90° left-rotated in Figure 3). A longer CCC pathway is not possible according to our current printability limitations in the MAM machine so that the current pathway is the longest way for the proposed circular CCC design. Moreover, the distance between the lateral surfaces of the circular CCCs and the surface of the polymer part is also determined regarding printability. For example, distances k and l are both 3 mm while the distance m is 5 mm in the current design (Figure 3). The distances k and l are the minimum possible distance values between the CCC lateral surface and the polymer part surfaces due to the printability concerns. However, the distance m is decided according to the thermal behaviors of the polymer product. That is, if the distance m becomes smaller than 5 mm, the CCC brings an extra cooling load that achieves a shorter cooling time but may result in high-temperature non-uniformity. According to the MAM machine type and polymer specifications, the distance values can vary. 

### 3.3. Serpentine CCC-Integrated Mold Geometry

**Figure 4:**
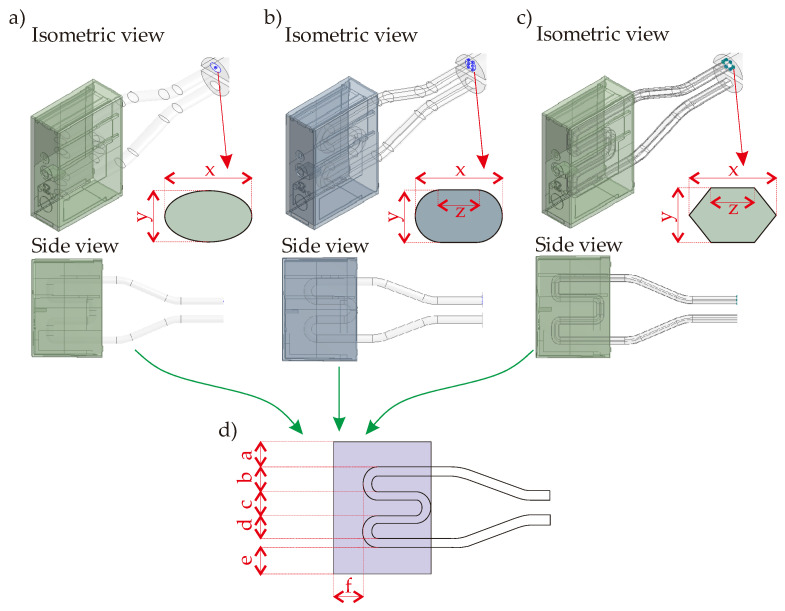
3D views of the serpentine CCCs: (**a**) elliptical, (**b**) elongated, (**c**) triangular, and (**d**) dimensions of the serpentine CCC in the mold geometry.

To increase the channel length in the mold geometry, the circular CCC is transformed to serpentine CCC designs by changing the cross-section of the channel. In the circular CCC, the maximum length is obtained via a U-shaped pathway. However, changing the circular cross-section to (i) elliptical, (ii) elongated, and (iii) triangular cross-sections allow increasing the pathway in the mold geometry; hereby, the CCC pathway is able to be created like serpentine instead of the U-shape pathway. Serpentine CCC has a longer length; hence, the coolant can be circulated for a longer time in the mold compared to the circular CCC. The longer channel pathway can increase the cooling performance but also cause the pressure drop in the channel. Figure 4 illustrates the serpentine CCC designs below.

All cross-sectional profiles of the serpentine CCC designs are defined with dimensions x and y as shown in Figure 4. In this study, x and y values are defined as 2.5 and 1.5 mm, respectively. That is to say, the x value (Figure 4) is the same as the D value of the circular channel (Figure 3), but y value (Figure 4) is smaller. Since the y value is smaller than the D value of the circular channel, the channel length is able to be designed as a serpentine type without exceeding the printability limits of the existing MAM machine. However, the smaller value may negatively affect the thermal performance so that the computational simulations will particularly point out this concern. Moreover, the elongated and the triangular types have an additional dimension, dimension z, with the value of 1 mm in Figure 4. Besides, Figure 4d shows various distances of the serpentine channel. Thanks to more flexibilities in the printing process, the distance between the CCC and the frontal surface of the polymer part, dimension f, is 3.5 mm that is smaller than the corresponding distance in the circular CCC (distance m, Figure 3). The smaller distance can bring positive (shorter cooling time) and negative (higher temperature non-uniformity) during the cooling process so that the results will be discussed in the following sections. The distances a–e are 3.5 mm for serpentine CCC types.

### 3.4. Tapered CCC-Integrated Mold Geometry

**Figure 5:**
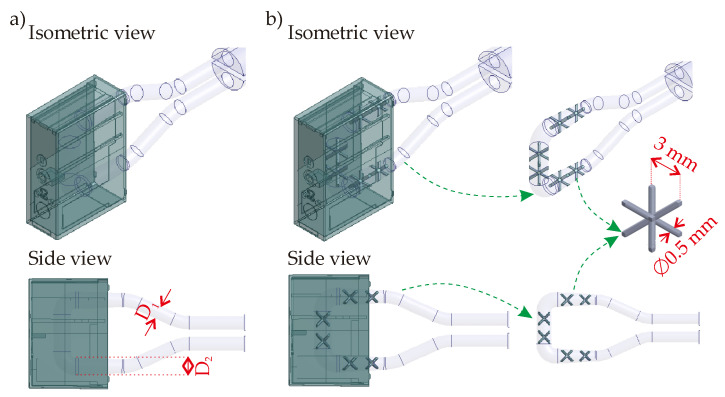
3D views of the tapered CCCs: (**a**) tapered and (**b**) BCC-integrated tapered types.

Another CCC type is proposed as the tapered design. The tapered CCC design is developed from the circular CCC design by changing the diameter to a greater value in the mold geometry as shown in Figure 5.

The tapered CCC is presented in Figure 5a. The initial diameter value, $D_{1}$, has a similar range with the D value of the circular CCC, between 2.1 and 2.5 mm. Then, the diameter is increased to $D_{2}$ value that has a range of 3.0–4.0 mm. By means of the increment, we aim to better manage the pressure drop in the CCC while achieving a shorter cooling time and more uniform temperature distribution. Furthermore, we consider further performance enhancement options like integrating lattice structures in the CCC as the MAM process allows us to create additional flexibilities. As shown in Figure 5b, six body-centered cubic (BCC) lattice structures are added to the channel. Hereby, the lateral surfaces of the BCC lattices will improve the heat transfer via convection and have the potential to achieve a shorter cooling time. On the other hand, the BCC lattices will behave like barriers against the coolant flow, which may result in pressure drop increments. To better understand the thermal performances of the proposed designs, computational models are presented in the next section. 

## 4. Computational Model

**Figure 6:**
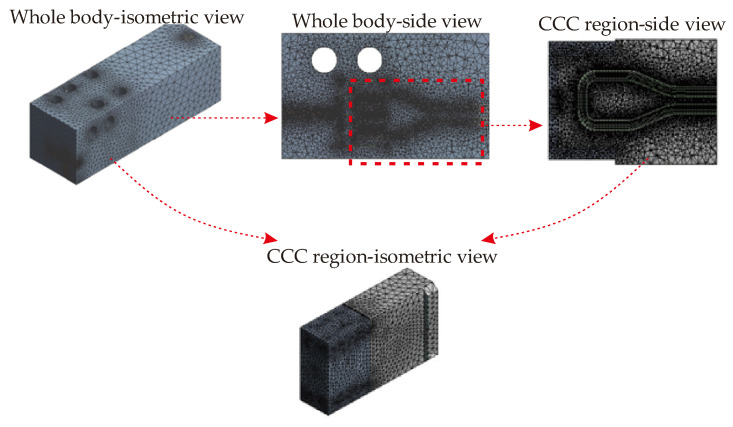
Mesh views of the conformal cooling channels via isometric and side views.

Computational simulations are performed using the main mold body shown in Figure 2a. However, the main focus is the conformal cooling-related parts that are explained in detail in Figure 3, Figure 4 and Figure 5. The current process is a three-dimensional (3D) transient heat transfer process so that the solution of mass, momentum, and energy equations must be clarified as presented in Equations (1)–(3), respectively,

(1) $$\nabla \cdot \vec{v}=0$$

(2) $$\rho \left(\frac{\partial \vec{v}}{\partial t}+\vec{v}\cdot \nabla \vec{v}\right)=-\nabla p+\mu \nabla^{2}\vec{v}$$

(3) $$\rho C_{p}\left(\frac{\partial T}{\partial t}+\vec{v}\cdot \nabla T\right)+\nabla \cdot \left(-k_{c}\nabla T\right)=0$$

where $\vec{v}$ and ρ is the velocity vector and the density of the coolant (water, t is time, p is pressure term, μ is the dynamic viscosity of the coolant (water), $k_{c}$ is the thermal conductivity of the coolant, and $C_{p}$ is the heat capacity. It is worth noting that the current model does not take the polymer flow into the account so that its control volume starts with the transferred heat from the polymer to the mold body. In the case of polymer flow considerations, viscoelasticity-related terms of relevant polymer flow must be added to the governing equations above. Besides the computational solution of the coolant in the mold, there is also a transient heat transfer inside the mold (stainless steel) body via conduction. Thus, it is solved using the energy equation given in Equation (4),

(4) $$\rho_{m}C_{p_{m}}\frac{\partial T}{\partial t}+\nabla \cdot \left(-k_{m}\nabla T\right)=0$$

where $\rho_{m}$, $C_{p_{m}}$, and $k_{m}$ are the density, heat capacity, and thermal conductivity of the mold body. After defining the governing equations required for solutions of the computational model, the meshing process is completed via tetrahedral mesh that minimizes the number of mesh compared to other types. The mesh view of the solution model is shown in Figure 6.

**Figure 7:**
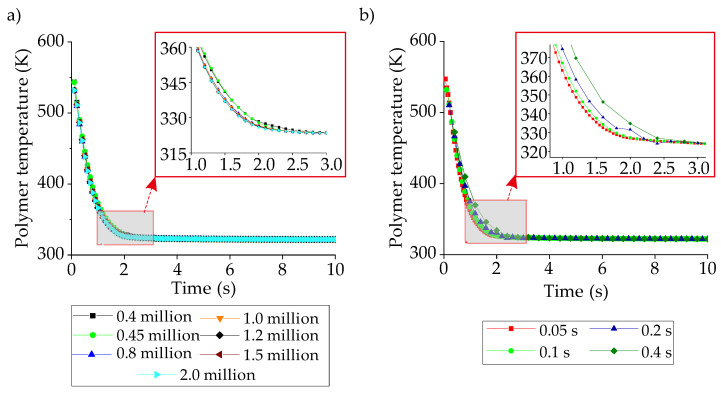
Independency of (**a**) mesh structure and (**b**) time-step.

Even though the whole body (Figure 2a) is used in the meshing step, we focus on the CCC region as illustrated in Figure 6. For mesh independence, seven different mesh sizes are investigated between 0.4 million to 2 million. Also, four different time-step values are compared for the time-step independence. Figure 7 projects the independence check for meshing and time-step.

The mesh independency results show that the mesh size of 1.2 million has very close trends with the mesh sizes of 1.5 and 2 million so that the independence is assumed to be achieved at the mesh size of 1.2 million. The time-step independence is provided with the time-step of 0.1 s as it coincides with the time-step of 0.05 s. Since all the boundary and initial conditions are the same for different types of CCCs, the same time-step value and similar mesh sizes are used in all simulations. The boundary conditions are; (i) the coolant inlet and outlet conditions are velocity inlet and pressure outlet, respectively, (ii) the mold surfaces contact with the plastic are defined with the coupled boundary conditions, (iii) the walls of the CCCs inside the mold geometry are defined with the convective boundary conditions, (iv) the SIMPLEC algorithm is used for the pressure-velocity coupling, and (v) the energy and momentum equations are discretized using the second-order upwind scheme. 

The thermal model aims to measure three objectives that are (i) cooling time, (ii) temperature non-uniformity, and (iii) pressure drop. Using the computational simulations, the cooling time is determined as the time from the injection temperature (543 K) to the ejection temperature (323 K). The temperature non-uniformity is the standard deviation of the plastic temperature as the surface as defined in Equations (5) and (6) below,

(5) $$\sqrt{\frac{\sum_{i=1}^{n}{\left(T_{i}-T_{0}\right)}^{2}}{n}}$$

where $T_{i}$ and $T_{0}$ are the cell temperature value (at each facet) and the mean temperature value, respectively, whilst the term n is the number of facets in the model,

(6) $$T_{0}=\frac{\sum_{i=1}^{n}T_{i}}{n}$$

Regarding Equations (5) and (6), a smaller standard deviation means that the non-uniformity is smaller because the temperature distribution is better. Therefore, the main purpose is minimizing the temperature non-uniformity whereas the minimizations of the cooling time and pressure drop are aimed as well. The pressure drop is calculated between the pressure values at the inlet and outlet regions.

The last step of the completion of the computational model is the experimental verification. The modelled cooling time of the straight-drilled channel was compared against experimental data of the straight-drilled channel obtained previously (as a cooling time of 7.0 s). An average relative difference of 7.14% was deemed acceptable for our 3D transient heat transfer studies. Apart from the computational thermal model, the mechanical strength can also be analyzed via computational mechanical simulations. Since the mechanical strengths were analyzed for the proposed mold geometry in our previous works [22,26,27], we do not mention them in this work.

## 5. Metamodel Development

**Figure 8:**
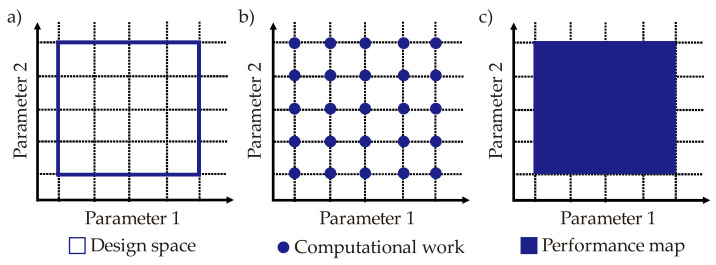
Illustrations of the metamodel development; (**a**) design space, (**b**) computational simulations, and (**c**) performance map.

Computational simulations provide numerical performance output for the objectives (cooling time, temperature non-uniformity, pressure drop). Each computational effort presents numerical results for a specific parameter set (e.g., a single value for each coolant temperature, volume flow rate, and diameter (or cross-sectional dimensions)). However, it is a fact that parameter sets create a design space for each CCC design so that a huge effort is required to define the general characteristics of the cooling performance (according to the defined objectives) between the lower and upper boundaries of the design space. Lower and upper boundaries can be defined as the minimum and maximum values of parameters, but multiple parametric sets should be defined with quantitative data using computational simulations. Hence, a designer (engineer, operator, etc.) who is fully or partially responsible for the entire process in Figure 1, should decide the number of computational solutions to define multiple parametric set points in the design space. There is a trade-off at this point; a higher number of computational simulations provides more quantitative data in the design space while it also results in higher computational time, cost, and labor. To balance the pros and cons, metamodels are widely used in this step. That is to say, several numbers of computational simulations are performed and thereby sufficient number of quantitative data can be obtained in the design space. After that, metamodel approaches are used to generate the overall performance trends (characteristics) of the objectives in the design space. To this end, a performance map for each objective can be created in the design space and the numerical values of the performance map can be used as the initial solution matrix during the multi-criteria decision-making step. Figure 8 illustrates the development of the metamodel from the design step to the final performance map using an imaginary scenario with two parameters for an objective. It is worth noting that this study has 3D design spaces (via three parameters; coolant temperature, volume flow rate, diameter/cross-sectional dimensions) for three different objectives which are pressure drop, cooling time, and temperature non-uniformity. 

For the circular and serpentine CCC designs, the metamodel was developed using MATLAB traditional interpolation approach [22,27] whereas the metamodel of the tapered (and BCC-integrated tapered) CCCs was developed via both MATLAB traditional interpolation and the neural network [23].

## 6. Multi-Criteria Decision Making

Multi-criteria decision making is a crucial step for selecting the most convenient option of the proposed CCCs as there are three different objectives. All objectives are aimed at their minimum values, but it is a fact that their performance maps generated by metamodels might not be in parallel to one another. That is, a continuous change in a target parametric set can provide decreasing trends for an objective while resulting in an increment for another objective. The multi-criteria decision making allows us to determine the most convenient case amongst different performance trends of the objectives. There are several approaches for multi-criteria decision making like using graphic tools, interpolative approaches, etc. Optimization is a preferred technique for decision making in the plastic injection industry [28,29], and its multiobjective procedure makes the decision making a multi-criteria framework. In this study, we prefer using the multiobjective optimization approach using the MATLAB Optimization Toolbox. 

The multiobjective optimization procedure is solved via Pareto frontier plot that consists of all the objectives between their lower and upper bounds. After creating a performance map for each objective, the objectives are brought together to quantitatively define the feasible solution region, which simply means the accumulation region of the parametric sets according to the lower and upper bounds of the objectives. Regarding the purpose of optimization (minimization or maximization) and the defined weightage values, the most convenient optimal point(s) can be found. In this study, we have three objectives so that the feasible solution region is a 3D region that is limited by the minimum and maximum values of the calculated objective values. As the main purpose is minimization for all objectives, we focus on the best trade-off point that is defined by aiming at the minimum values of objectives. It must be noted that the multiobjective optimization solution becomes easy to handle if the performance maps of objectives have parallel trends to one another. However, in the case of opposite performance trends (like in this study), the decreasing trends of objective results in an increasing trend for another objective so that the weightage value becomes critical during the decision-making step. Following this fundamental, the multiobjective optimization procedure is applied in three main steps. The first step is generating the borders of the feasible solution region via the Pareto frontier plot in the MATLAB toolbox. This is an automated process of the MATLAB after giving input of the objective functions, constraints, lower & upper bounds. The genetic algorithm recognizes the performance map of each objective as the initial population; and then, creates the feasible solution region via the Pareto frontier. The second step is normalizing the objective function values by using the utopia and pseudo-nadir points as shown in Equation (7),

(7) $$f^{*}\left(x\right)=\frac{f\left(x\right)-f^{U}\left(x\right)}{f^{PN}\left(x\right)-f^{U}\left(x\right)}$$

where f(x) is the value of the objective in the Pareto frontier; $f^{*}\left(x\right)$ is the normalized value of f(x); $f^{U}\left(x\right)$ and $f^{PN}\left(x\right)$ are the utopia and pseudo-nadir points of the Pareto frontier. The utopia point, $f^{U}\left(x\right)$, is the value point in the plot where all objectives have their minimum values. However, this point is not in the feasible solution region because there is no feasible solution for the optimization when all the values go to their minimum values since they have opposite trends in one another in the current problem. Contrary, the pseudo-nadir point means the point of the most undesired values (maximum values in this problem) in the feasible solution region. It is noted that the $f^{PN}\left(x\right)$ stays in the feasible solution region unlike the $f^{U}\left(x\right)$. Also, the nadir point, $f^{N}\left(x\right)$, which is mentioned neither in Equation (7) nor in the general solution, is the point where the maximum values of objectives outside of the feasible solution region. The normalization procedure is applied via $f^{U}\left(x\right)$ and $f^{PN}\left(x\right)$ in this work. However, in case of maximization purpose for all objectives, the term $f^{N}\left(x\right)$ could have been considered in the normalization step. 

Since all objectives aim minimization in the solution procedure, the weightage of objectives is significant. Hereby, we are able to determine the best trade-off point using the Pareto frontier plot data. After completing the normalization, the weightage values are defined as $\omega_{ct}$, $\omega_{tn}$, and $\omega_{pd}$ for the objectives of cooling time, temperature non-uniformity, and pressure drop, respectively. The impact of weightage is calculated using the weighted-sum (WS) approach as shown in Equation (8),

(8) $$WS=f^{*}{\left(x\right)}_{ct}\cdot w_{ct}+f^{*}{\left(x\right)}_{tn}\cdot w_{tn}+f^{*}{\left(x\right)}_{pd}\cdot w_{pd}\;\mathrm{where}\;w_{ct}+w_{tn}+w_{pd}=1$$

The WS is one of the weightage analysis approaches and we prefer it in this study. However, other approaches like weighted-product (WP) can be applied as well. The choice of the weighting approach depends on the decision-maker.

## 7. Metal Additive Manufacturing Process

**Figure 9:**
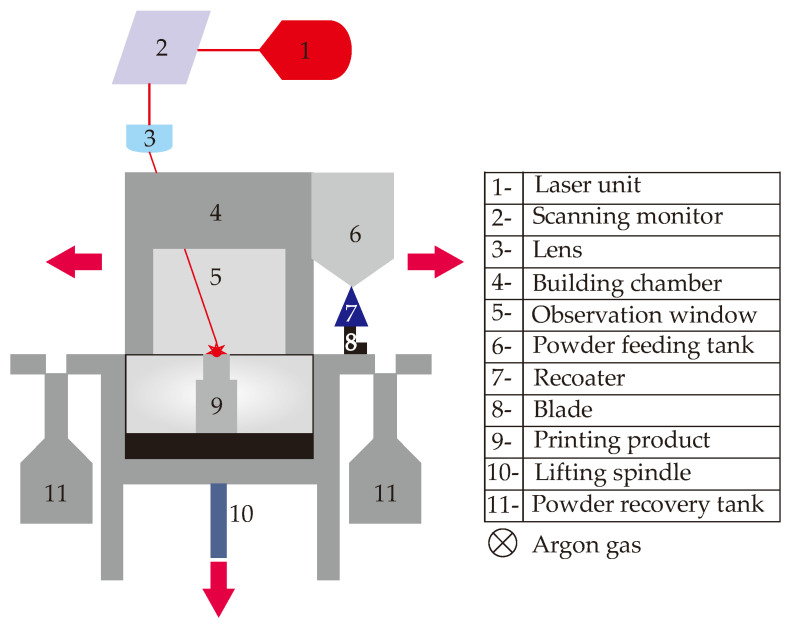
Simplified schematic of the DMLS machine.

The metal additive manufacturing process is performed using the Direct Metal Laser Sintering (DMLS) approach that is one of the most economically feasible MAM techniques thanks to its benefits like stable manufacturing environment, smaller building platform that reduces the metal powder waste, and smaller average powder size that achieves the printability of smaller geometric feature size. The DMLS uses the focused laser beam in order to melt the micron-sized metallic powders, which is maraging steel MS1 powder in this work. Chemical and mechanical properties of MS1 powder are obtained by manufacturer dataset and shared in Table 1. DMLS provides layer-by-layer laser scanning; hereby, the stack of two dimensional (2D) layers form the 3D product. The DMLS process is employed with the commercial M100 (EOS GmbH) machine that consists of the laser system, building module, and powder collection system (for unmelted powders). The simplified schematic of the DMLS process is projected in Figure 9.

The DMLS process performance depends on various variables such as laser system parameters, laser scanning method and the layout of the printed molds. The laser system parameters are related to the energy input to the machine, and it affects the product quality (number 9 in Figure 9). Laser energy density is a useful performance indicator to quantify the energy input to the DMLS machine as shown in Equation (9),

(9) $$E=\frac{P}{u\cdot h\cdot d}$$

where E is the laser energy density (J/mm^3^), P is the laser power (W), u is the scan rate (mm/s), h is the scan spacing (mm), and d is the layer thickness (mm). To show the impacts of the laser system parameters on the printing quality, we performed five different parametric scenarios by keeping the layer thickness and the scan spacing at constant values of 0.02 mm and 0.06 mm, respectively; and varying the laser power and the scan rate. Since the BCC-integrated tapered CCC is the most challenging design amongst others, we use that design when we are investigating the DMLS process performance. Regarding the variables (laser power and scan rate), Table 2 presents these five different scenarios below. 

**Figure 10:**
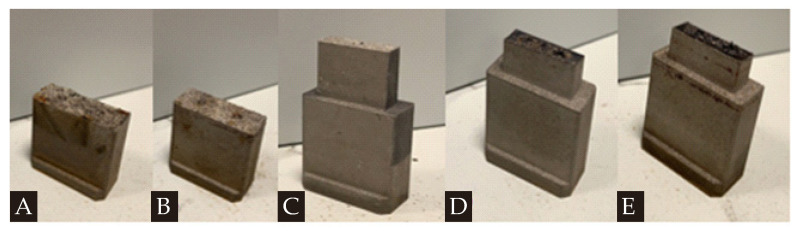
DMLS process for five different printing scenarios of the BCC-integrated tapered CCC.

Following the given five different scenarios, a preliminary printing process is applied for the BCC-integrated tapered CCC design as an observational sampling study. Figure 10 shows the printing results for these scenarios.

**Figure 11:**
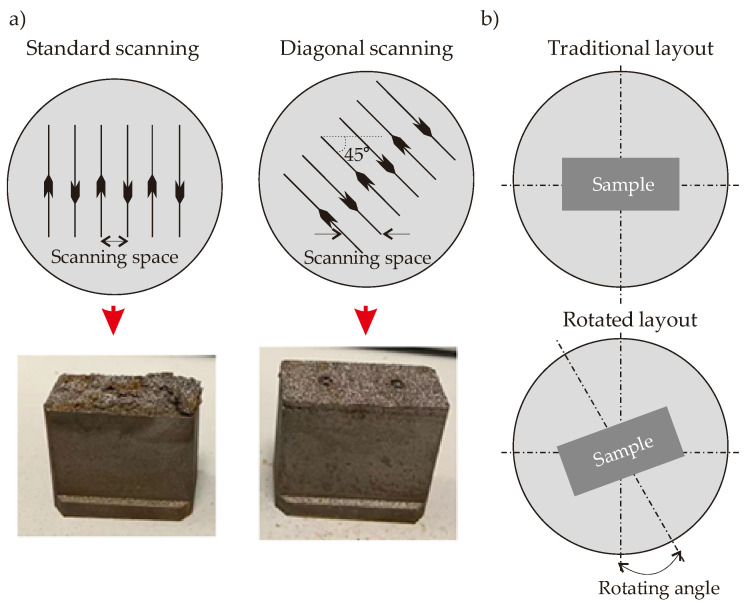
DMLS process parameters; (**a**) scanning method and (**b**) sample layout.

It is seen that the printing-related failures are observed for all scenarios except for Scenario C (Sample Code C in Table 2). Therefore, in this study, we select the laser system parameters of Scenario C. It is worth mentioning that different parametric values can also be tried and preferred (if they work). Since we compare five different scenarios, we select the only scenario that reliable works. However, it is worth noting that other scenarios may give effective results with different printing facilities, but it was clear that they were not convenient for our printing facilities/environment. Apart from experimental trial-and-error efforts, another way to determine the convenient printing parameters is comparing the laser energy density with the minimum amount of heat requirement for sufficient melting phenomena. This is a quantitative comparison approach based on the thermal energy balance and independent of printing machine-related issues that can be encountered during the printing process; but it is a basic way to understand the efficacy of the selected printing parameters. More details on this procedure can be found in ref. [30]. The laser scanning method also matters for the printed quality so that we focus on two well-known scanning methods that are standard and diagonal scanning. It is known that both scanning techniques apply a high-power focused laser beam in order to scan the perimeter of the required cross-section area at first. The main difference between these two techniques is the diagonal scanning pattern keeps a constant angle of 45° to on the *x*- and *y*-axis whilst all patterns are parallel to the *x*- (or *y*-) axis. That is, two techniques follow different patterns to scan the internal area. Like in the laser system parameters, the BCC-integrated tapered CCC is considered as the printing part in a preliminary printing process of another observational sampling study. Figure 11a displays the simplified patterns of two different scanning methods with the printed parts. Besides the scanning method, the layout of the part is also critical. The traditional layout is placing the sample in parallel to the *x*- (or *y*-) axis on the building platform. During the preliminary printing of the BCC-integrated tapered CCC model, the traditional layout successfully worked so that we prefer the traditional layout. However, in case of failures, the placed sample can be rotated to keep a constant angle to the *y*-axis (mostly). Figure 11b illustrates the traditional and rotated layouts.

As shown in Figure 11a, serious delamination occurred in the part when the standard scanning method is preferred while there is no significant delamination in the diagonal scanning technique so that the diagonal scanning is preferred in our printing works in this study. The reason behind the formed delamination is the large thermal stress vectors. The large thermal stress results in protruding layers which damage the recoater & blade (numbers 7 and 8 in Figure 9) and lead to dendritic metals on the subsequent layer.

**Figure 12:**
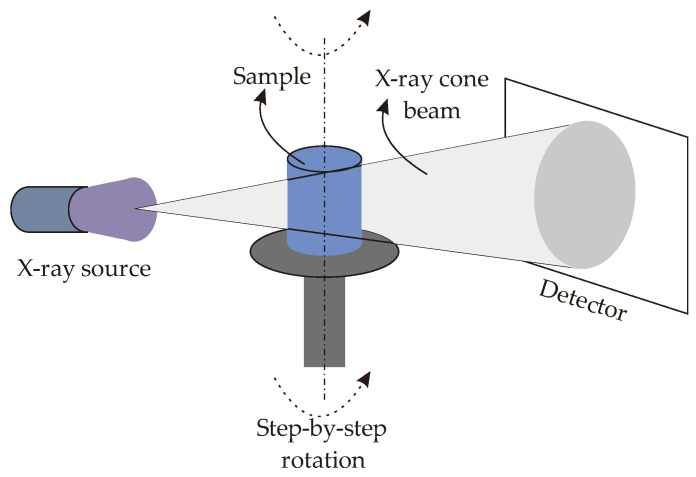
Simplified schematic of the X-ray CT system.

The MAM process quality is monitored using the X-ray Computed Tomography (X-ray CT) approach. As the CCCs are inside of the metal printed body, X-ray CT provides an effective and reliable scanning environment in order to measure the dimensions of the printed CCCs (e.g.; MAM process of microchannels in aluminum printing [31]). X-ray CT is a non-destructive (NDT) technique that can be summarized as (i) emitting a cone-beam of X-rays through the printing sample, (ii) generating projections based on the intensity of X-rays falling on the detector, (iii) calculating 2D CT images from projections, and (iv) stacking multiple 2D CT images into the 3D ones. The principle of the X-ray CT measurement is shown in Figure 12.

The X-ray source is operated with the current and voltage values of 0.7 μA and 450 kV, respectively; where the scanned image has the spatial resolution of 0.5 mm. A constant rotation step of 0.9° is used as the acquisition interval. Hereby, we obtained four hundred (400) 2D CT images and these images were used to build the 3D CT image using the commercial software of VGStudio MAX 3.4 (Volume Graphic GmbH, Heidelberg, Germany). Thanks to the 3D CT image, we can visualize the CCC pathways in the printed mold geometries. 

To quantify the printing quality of the products, we define some locations/regions on the designed/printed product; then, we compare the designed dimension and the measured dimension. Hereby, we can understand the occurred errors during the pre-printing (e.g., model transition to the MAM environment), printing (e.g., printing parameters), and post-printing (e.g., dimensional errors) processes. The error analysis can also provide us with useful information for further process operations. The relative error is defined in Equation (10),

(10) $$\in =\frac{\left|x_{M}-x_{D}\right|}{x_{D}}$$

where $x_{M}$ and $x_{D}$ are the measured and the designed dimensions at a specific location in the printing product, respectively. In this study, we calculated error according to multiple locations in the printed product for more reliable error analysis. Details are explained in Section 8.3.

## 8. Results and Discussions

### 8.1. Computational Simulations and Performance Maps

#### 8.1.1. Circular CCC

**Figure 13:**
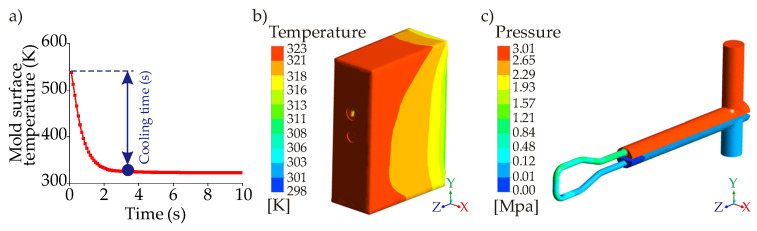
Representative CFD case of a circular channel (D = 2.1 mm, coolant temperature = 298 K, volume flow rate = 10 L/min) results; (**a**) cooling time, (**b**) temperature distribution, and (**c**) pressure drop.

Results of the computational simulations and metamodels (performance maps) are evaluated according to the main objectives that are the cooling time, temperature non-uniformity, and pressure drop. Figure 13 represents the results of a case of a circular channel for three different objectives. 

**Figure 14:**
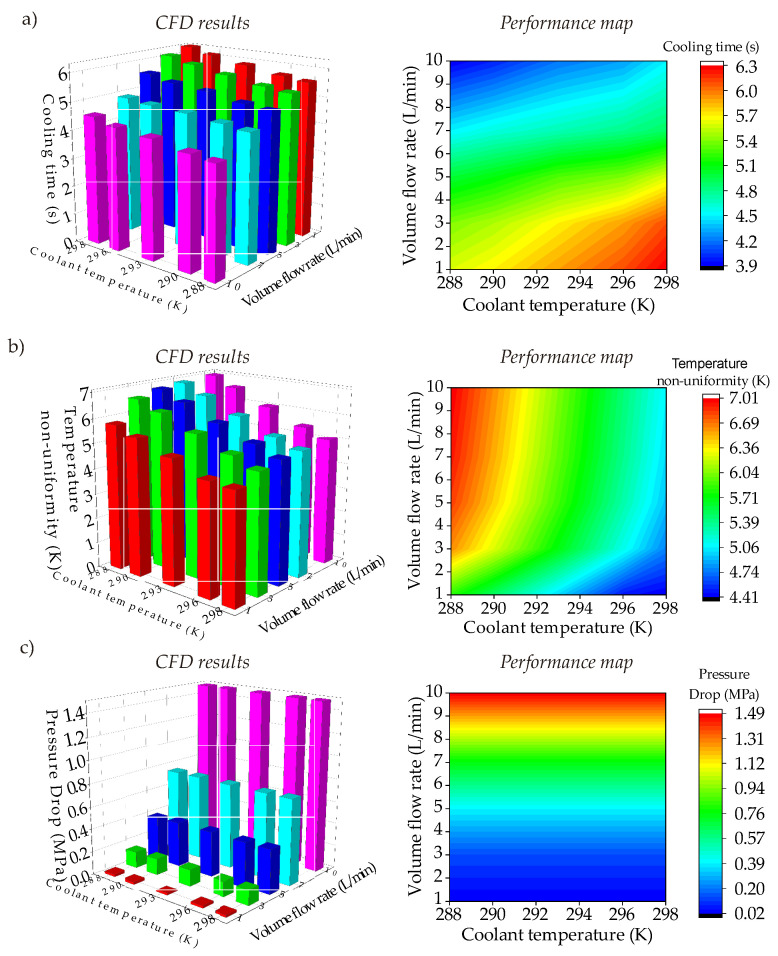
Computational results and performance maps for the circular CCC with D = 2.1 mm; (**a**) cooling time, (**b**) temperature non-uniformity, and (**c**) pressure drop.

Following the outcomes of the computational simulations, performance maps generated by metamodels. As mentioned in Section 5 (metamodel development), each CFD result is marked in the design space first; then, combining the increasing/decreasing trends of the computational simulations, performance maps are generated. Figure 14 shows the CFD results and corresponding performance maps for the main objective functions of the circular CCC with D = 2.1 mm. The whole data of circular CCCs from D = 2.1 mm to D = 2.5 can be seen in Table A1. Regarding Figure 14, it is seen that the cooling time reaches its minimum value of 3.9 s at the coolant temperature of 288 K and the volume flow rate of 10 L/min, which is an expected outcome due to the high-volume flow rate and low coolant temperature. Compared to the cooling time performance of the straight-drilled channel, which is 7.0 s (see Section 4), the circular CCC decreases the cooling time by 44.3%. On the other hand, the maximum cooling time value is 6.3 s that belongs to the parameter set of the cooling temperature of 298 K and the volume flow rate of 1 L/min. This shows that the circular CCC with D = 2.1 achieves the cooling time decrement at least by 10% compared to the straight-drilled channel. However, as the main point of this study, the cooling time is not the primary objective; it should be considered with other two objectives: temperature non-uniformity and pressure drop. When the temperature non-uniformity values are seen in Figure 14b, it is seen that the maximum temperature non-uniformity is 7.01 K at the coolant temperature of 288 K and the volume flow rate of 10 L/s, which is exactly the same parameter set of the minimum cooling time data. The minimum temperature non-uniformity is 4.41 K at the coolant temperature of 298 K and the volume flow rate 1 L/min. When the cooling time and the temperature non-uniformity trends are evaluated together, it is clearly seen that the difference between the minimum and maximum values are more significant for temperature uniformity and the trends of cooling time & temperature non-uniformity are opposite to each other. This opposition supports our motivation for selecting the multi-criteria decision-making according to multiple objectives. Besides the circular CCCs at D = 2.1 mm, Table A1 presents the results for other diameters. The increase of diameter from 2.1 mm to 2.5 decreases the minimum cooling time from 3.9 to 3.6 s while grows the maximum temperature non-uniformity from 7.01 K to 7.29 K. That is, the increment rate of temperature non-uniformity is 3.9% while the decrement rate of cooling time is 7.7% from D = 2.1 to D = 2.5 mm. Differently from others, the pressure drop (Figure 14c) inherently is affected only by volume flow rate for a constant diameter. For D = 2.1 mm, the pressure drop increases by 1. 45 MPa from the volume flow rate of 1 L/min to 10 L/min. When the diameter increases from 2.1 to 2.5 mm (Table A1), the pressure drop decreases by 59% (from 1.49 MPa to 0.61 MPa) at the volume flow rate of 10 L/min. It is worth noting that the decrement rate of pressure drop becomes smaller by decreasing of volume flow rate. For example, at the volume flow rate of 1 L/min, the decrement is very small, only at decimal levels.

#### 8.1.2. Serpentine CCC

**Figure 15:**
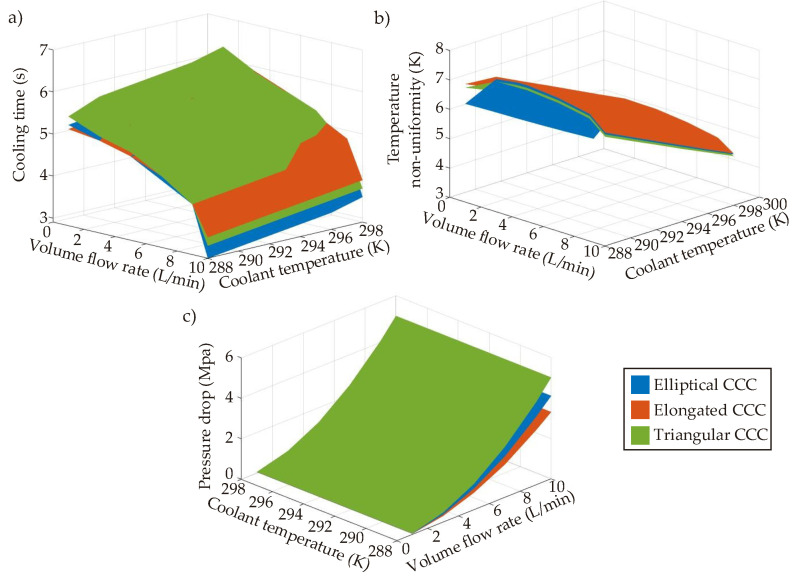
Performance maps for the serpentine CCCs; (**a**) cooling time, (**b**) temperature non-uniformity and (**c**) pressure drop.

Serpentine CCCs are designed to provide smaller temperature non-uniformity with shorter cooling time compared to the circular CCCs. Figure 15 presents the performance maps of the serpentine channels according to the cooling time, temperature non-uniformity, and pressure drop. All serpentine CCC types show similar trends in the design space. Like the circular CCCs, the minimum cooling time is seen at the coolant temperature of 288 K and the volume flow rate of 10 L/min. Amongst others, the elliptic CCC has the shortest cooling time with 2.9 s; followed by triangular CCC with 3.2 s and elongated CCC with 3.4 s. The maximum cooling time is 6.0 s, 6.1 s, and 6.3 s for the elliptic CCC, elongated CCC, and triangular CCC, respectively, at the coolant temperature of 298 K and the volume flow rate of 10 L/min. Between the minimum and maximum values, the elliptic CCC has the minimum cooling time variation and it is followed by the elongated CCC and triangular CCC. Similarly, the minimum variation between the minimum and maximum temperature non-uniformity values belongs to the elliptic CCC and it is followed by the triangular CCC and elongated CCC. Also, the minimum temperature non-uniformity is seen for the elliptic CCC with the temperature non-uniformity value of 3.77 K. For the elongated CCC and the triangular CCC, the minimum temperature non-uniformity values are 5.08 K and 4.99 K, respectively. This shows that there is a significant difference between the minimum temperature non-uniformity values of different serpentine CCCs. The whole CFD results of the serpentine CCCs are shown in Table A2. 

Apart from the cooling time and temperature non-uniformity trends, the pressure drop trends are also projected in Figure 15 and listed in Table A2. Like the circular CCCs, the pressure drop is only affected by the volume flow rate; the increase of the volume flow rate significantly raises the pressure drop. The pressure drop changes are not significant at the low volume flow rates; however, they become important by raising of the volume flow rate. At the highest volume flow rate, 10 L/min, the pressure drops are 3.31 MPa, 4.11 MPa, and 5.02 MPa for the elongated CCC, elliptical CCC, and triangular CCC, respectively. Following the CFD results, it can be said that the elliptical CCC is the most convenient one from the viewpoints of cooling time and temperature non-uniformity. In fact, the elliptical CCC provides even better performance than the circular CCCs with respect to the cooling time and temperature non-uniformity although the difference is not significantly high. This shows that changing the lateral surface from circular to elliptical can improve the performance since the elliptical surface can have a better interaction with the mold surface through the convective heat transfer. On the other hand, the serpentine CCCs result in significantly high pressure drops compared to circular CCCs, and this makes the serpentine CCCs less preferred. Therefore, in this work, the integration of BCC lattices into the tapered CCCs is applied with the circular cross-section profiles.

#### 8.1.3. Tapered CCC

**Figure 16:**
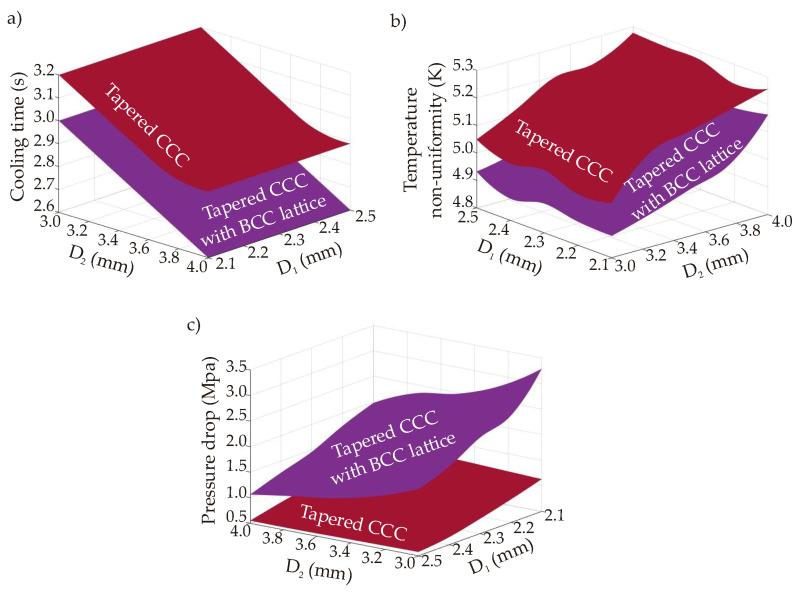
Performance maps for the tapered CCCs; (**a**) cooling time, (**b**) temperature non-uniformity and (**c**) pressure drop (at the coolant temperature of 298 K and the volume flow rate of 10 L/min).

The tapered CCC aims to extend the lateral (internal wall) surface area in the mold geometry by changing the diameter from a smaller value to a higher value; hereby, a greater heat transfer surface can be obtained. Due to the advantages of the circular cross-section compared to the elliptical, elongated, and triangular CCCs, we prefer circular cross-section for tapered CCCs. Figure 16 shows the performance maps of the tapered CCC and the BCC lattice-integrated tapered CCC according to cooling time, temperature non-uniformity, and pressure drop. Also, the whole CFD results are given in Table A3.

The performance map of the cooling time infers that the cooling time is not affected by the circular channel diameter, D_1_ in Figure 5, as that part is away from the hot spots of the mold part, which interacts with the melt polymer. Only D_2_ value affects the cooling time trends because that part is directly in contact with the hottest spots and regions in the mold geometry. The minimum and maximum cooling time of the tapered CCC are 2.9 and 3.2 s, respectively; which shows that the tapered CCC design achieves better cooling time performance than the circular CCC. Compared to the circular CCC from D = 2.1 mm to D = 2.5 mm, the tapered CCC shortens the cooling time in the range of 29.3–35.6%. Also, compared to the straight-drilled channels, the cooling time is decreased by 58.6%. It is also seen that the BCC lattice integration provides further cooling time decrement as the BCC lattice surfaces increase the heat transfer rate through the convection and conduction mechanisms. Compared to the tapered CCC, BCC integration decreased the minimum cooling time from 2.9 s to 2.6 s; which makes the cooling time decrement by 62.9% when it is compared with the cooling time performance of the straight-drilled channel. The temperature non-uniformity map projects that both D_1_ and D_2_ affect the temperature uniformity but the impact of D_2_ is inherently higher due to the same reason for the cooling time trends; D_2_ is very near the hot spots and regions in the mold geometry. Similar to the cooling time trends, the tapered CCC achieves smaller temperature non-uniformity with an average decrement rate of 2.4%. The decrement rate is small so that it can be assumed that there is no significant change in the temperature non-uniformity trends between the circular CCCs and the tapered CCCs although the tapered CCC achieves a shorter cooling time. When the BCC lattices are integrated into the tapered CCC, the decrement in the temperature non-uniformity becomes larger with the average decrement rate of 5.7% compared to the circular CCCs from D = 2.1 mm to D = 2.5 mm. Another effective point of tapered CCCs is the positive impact of the tapered design on the pressure drop. The difference between the minimum and maximum pressure drop values is not large and the average pressure drop of the tapered CCC is calculated 0.84 MPa. The pressure drop trends of the tapered CCC is smaller than the circular CCCs from D = 2.1 mm to D = 2.3 mm, but larger than the circular CCCs from D = 2.4 mm to D.2.5 mm. That is, the pressure drop trends of the tapered CCC is in the same range as the pressure drop trends of the circular CCCs. However, the integration of the BCC lattices increases the pressure drop since the lattice bodies result in additional flow resistance inside the channel. The pressure drop values of the BCC-integrated tapered CCC range between 1.06 and 3.29 MPa and the average pressure drop value is 1.91 MPa. These trends are larger than the pressure drop trends of the circular CCCs from D = 2.1 mm to D = 2.5 mm. That is, the BCC integration achieves better cooling time and temperature non-uniformity but results in higher pressure drop compared to both tapered CCCs and circular CCCs. Moreover, it is seen that the impact of D_1_ on the pressure drop is greater than the impact of D_2_ because the D_1_ directly connects with the main coolant inlet, which has a higher diameter than both D_1_ and D_2_ (see Figure 2), thus the biggest pressure drop occurs at that stage. 

### 8.2. Multi-Criteria Decision Making

**Figure 17:**
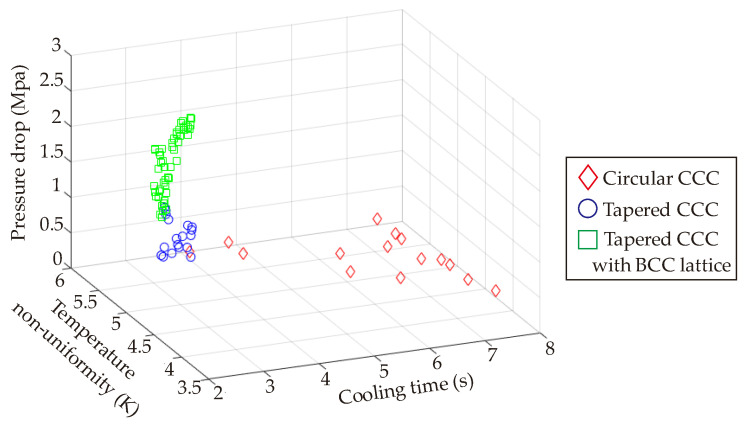
Pareto frontier plots of the circular CCCs and tapered CCCs.

Computational simulations and performance maps show that the tapered CCCs (both tapered CCC and the BCC-integrated tapered CCC) and circular CCCs provide preferable performances from the viewpoints of cooling time, temperature non-uniformity, and pressure drop. Selection of the convenient design should depend on the weights of the objectives in the multi-criteria decision-making procedure, but it is clear that the serpentine CCCs are less preferable. Considering all objectives, the multi-criteria decision-making procedure focuses on the multiobjective optimization & 3D printing of the circular CCCs and tapered CCCs together. Following the multiobjective optimization procedure in Section 6, Figure 17 projects the MATLAB-based Pareto frontier plots for the circular CCC, tapered CCC, and the tapered CCC with BCC lattices. The Pareto frontier plots of the circular CCC have a wider solution region compared to the tapered CCC and the BCC-integrated tapered CCC because the operating conditions of the circular CCC are defined in a broader range; in the ranges of 288.0 K–298.0 K and 1 L/min–10 L/min for the coolant temperature and the volume flow rate, respectively. On the other hand, since the tapered CCC studies only focus on the changes in D_2_ and D_1_, we performed their simulations at the constant values of coolant temperature (298 K) and the volume flow rate (10 L/min). That is to say, even though the Pareto frontier plots of the circular CCCs and tapered CCCs are shown in the same figure (Figure 17), their parameter sets are different so that we perform separate multiobjective optimization procedures for the circular CCCs and the tapered CCCs. The obtained Pareto frontier plots allow us to select the most convenient point according to our preferences. In this study, we put equal weights for all three objectives; namely, the weightage of each objective is 1/3 (~0.333 or ~33.3%). The weighted-sum approach in Equation (8) is applied with the same weightage values for all objectives; then, the best trade-off points are obtained for each CCC type; circular CCC, tapered CCC, and tapered CCC with BCC lattice. The best trade-off point of the circular CCC is found at the coolant temperature of 297.68 K, volume flow rate of 1.01 L/min, and diameter of 2.5 mm. This shows that the equal weightage in the multiobjective optimization procedure provides the best trade-off point at the values of the maximum coolant temperature, the minimum volume flow rate, and the maximum pressure drop values. Even though the cooling time reaches its minimum value at the minimum coolant temperature value, the combined impact of the temperature non-uniformity and the pressure drop trends on the best trade-off point makes the most convenient coolant temperature at 297.68 K. The best trade-off point of the tapered CCCs are determined according to the parameters of D_1_ and D_2_ without considering the coolant temperature and volume flow rate. At equal weights, the best trade-off is obtained at the maximum value (2.5 mm) for D_1_ and close to the maximum value (3.8 mm) for D_2_. That is to say, although high diameter values increase the temperature uniformity, they are found feasible as they minimize the pressure drop and cooling time (Figure 16). Also, diameter values (D_1_ and D_2_) of the best trade-off points of the tapered CCC and the tapered CCC with BCC lattices are the same but their corresponding cooling and pressure parameters (cooling time, pressure drop, temperature non-uniformity) are different as discussed in Section 8.1.1 and Section 8.1.3. It is worth noting that the obtained best trade-off points are for equal weights. In the case of non-equal weights, the best trade-off point values depend on the primary objective functions. 

### 8.3. Metal Additive Manufacturing of the CCCs

The completion of the multiobjective optimization process makes ready the MAM of the circular and the tapered CCCs. Even though the tapered CCCs have better cooling performance than the circular CCCs, the circular CCCs are also manufactured in order to see the printing quality for circular cross-section profiles. The serpentine CCCs are not considered as they do not provide promising outcomes compared to the circular and the tapered CCCs. The multiobjective optimization includes three objectives for both CCCs but two of these objectives are flow parameters. Only the diameter is the printing-related objective so that the MAM process is based on the diameter values regarding the best trade-off point values. Table 3 shows the target diameters for CCCs in the MAM process. 

**Figure 18:**
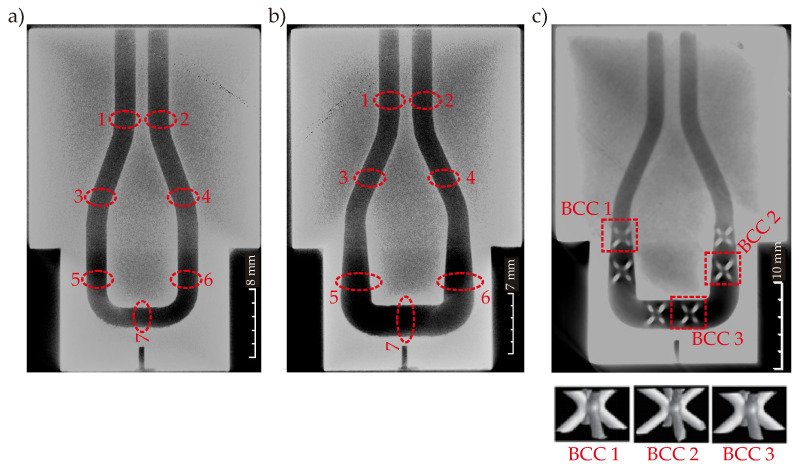
X-ray CT scan images of (**a**) circular CCC, (**b**) tapered CCC, and (**c**) tapered CCC with BCC lattice.

The MAM process is performed for all CCC types with multiple printing; each CCC type is printed at least three times following the printing parameters given in Table 2 (scenario C) and Figure 10 (scenario C). Since the CCCs are internal body features that cannot be viewed from outside, we use the X-ray CT scan for measuring the channel and BCC dimensions. Figure 18 shows the X-ray CT scan images of the circular CCC, tapered CCC, and tapered CCC with BCC lattice. 

The X-ray CT images show that the mold body has a good quality without undesired porous mediums. However, the channel dimensions are the most critical factors to determine the printing quality. Thus, seven different locations are defined in the mold and the channel diameters are measured at these seven locations for circular and tapered CCCs. Figure 18 shows the mentioned seven locations and three BCC lattices for the printing quality check. The measured diameters of the CCCs at seven different locations are presented in Table 4 and compared with the target diameter values.

Table 4 deduces that the calculated error for CCCs are lower than 5%, which shows that the printing is done in high-quality with an acceptable error. The results also show that the error is not affected by the integrated BCC lattices in the channel body. That is, integrating the BCC lattices into the channel body does not decrease the printing quality but inherently increases the printing time. The increase in printing time is directly related to the printing parameters. In addition, the X-ray CT scan images infer that the diameter change during the printing (tapered CCCs) does not affect the printing quality. Besides the printing check with the channel diameters, three BCC lattices are selected for measurement to see the printing quality of the BCC lattices in the channel body. The selected BCC lattices can be seen in Figure 18. For each BCC lattice, we measure four strut diameter values and calculated the maximum deviation by comparing it to the target strut diameter value of 0.5 mm. The measured dimensions are given in Table 5. 

The measured strut diameters imply that the maximum deviation values are the same for three different BCCs in the channel and the deviation value is very small compared to the target diameter value. Therefore, we can state that the printing parameters provide high quality even for small diameter values of BCCs and the printing process is successfully finalized regarding the target values obtained by the multiobjective optimization procedure. 

### 8.4. Limitations and Challenges

The entire MAM process of the CCCs shows that the CCCs can be successfully designed and manufactured by MAM tools to replace the traditional straight-drilled channels in plastic injection technology. The flexibilities of the MAM process provide numerous advantages to better manage the plastic product quality thanks to a better design and optimization process of the plastic injection molds. Using applicable printing parameter sets, the product can be manufactured with high quality. Besides all these benefits, there are some practical limitations and relevant challenges, which can depend on the procedures and processes of the design, decision, and printing. Below, they are explained one by one. 

The main limitation of the design (including CAD design) is the distances between the channels and the mold surfaces. It is a well-known fact that the cooling time can be decreased when the distance between the CCC and the mold surface becomes smaller. However, two challenges appear; (i) temperature non-uniformity can increase, and (ii) there are structural limits (regarding the mechanical performance) that do not allow us to design the CCC very near the mold surface. Details of this challenge can be found in ref. [21].

The simulation process is usually limited by the computing performance. Unlike the straight-drilled channels, 2D simulations cannot represent the thermal and flow behaviors of the cooling process because the CCCs are complex designs with a 3D perspective (see ref. [32]). Also, the cooling process is a transient process that cannot be analyzed very well via steady-state simulations. Therefore, the simulation process requires high-performance computing tools to collect the simulations results in a short time. In the case of long simulation times, the whole process can take a long time, which decreases its economic feasibility. 

Although metamodels bring a “bridge” solution between the computational simulations and decision-making process (e.g., multiobjective optimization) by decreasing the required number of simulations, the fundamentals of the applied metamodel should be known very well to avoid potential errors and deviations. Interpolations, basic neural network structures, and Gaussian approaches can generate reliable and accurate metamodels for products with a low number of design variables. However, for the products with a high number of design variables, the metamodels can result in undesirable and unplanned deviations that can affect the accuracy of the performance maps; thereby, the decision-making procedure gives inaccurate outcomes. To overcome this challenge, more complex neural network structures or hybrid approaches (see refs. [24,33,34]) should be applied instead of basic neural networks or interpolation algorithms. Also, like computational simulations, the computing time and cost are limits for the metamodel process, especially for products with a high number of design variables. 

The multiobjective optimization procedure is an efficient and useful way to perform decision-making among multiple objectives. The algorithm can easily handle multiple objectives with clarified lower and upper bounds. However, in the case of complex constraints that can also cause dynamic lower and upper bounds during the transient process, the multiobjective optimization procedure can be a complicated step. For example, some plastic injection molding processes have additional and non-continuous heating steps [35], or some injection molding processes are aimed for thin walls [36]. These applications are not parts of traditional plastic injection molding processes and they bring additional constraints that can affect determining the lower and upper bounds. In these applications, designers and decision-makers should be aware of the impacts of these additional challenges; otherwise, the Pareto frontier plots can be negatively affected by uncertainties. 

The last step of the entire process, the MAM of the plastic injection molds, has the main limitations from the viewpoints of the printing device and printing parameters. Different printing machines and printing parameters can present different printing qualities; therefore, the analysis of the printing parameters should be done with a parametric work and the best printing parameters should be chosen according to the process performance. It must be noted that the printing quality might not be the same for the multiple printing of the same product so that the comparative quality check should also be done. In this study, there are no significant quality differences between the printing parts. 

## 9. Conclusions

The presented study investigated the entire process of the metal additive manufacturing of the conformal cooling channels (CCCs) in plastic injection technology from the initial design step to the final printing step. A plastic injection mold product with multiple design parameters and three main objectives, which were cooling time, temperature non-uniformity, and pressure drop; was focused on computational design, simulations, metamodels, and optimization studies. Three different conformal cooling channel types were developed that were circular CCC, serpentine CCC, and tapered CCC. Compared to the traditional straight-drilled channel, the tapered CCC achieved to decrease in the cooling time by 58.6%. When BCC lattice structures were integrated into the channel body, the heat transfer via conduction and convection increased so that the cooling time decrement was obtained at 62.9%. The cooling time decrement via circular CCCs was 44.3%; however, the circular CCCs had lower temperature non-uniformity by 5.7% than the tapered CCC with BCC lattice whilst there was no significant difference between the temperature non-uniformity values of circular CCCs and tapered CCCs. Pressure drop trends inferred that the tapered CCCs and circular CCCs had very similar pressure drop values but the integration of BCC lattices increased the pressure drop to the average value of 1.91 MPa. Amongst others, the serpentine channels could not achieve satisfying cooling performances as circular CCCs and tapered CCCs as; also, they resulted in high-pressure drop. Thus, the multiobjective optimization study was performed with the circular CCCs and tapered CCCs. The best trade-off points were determined according to the equal weightage (33%) for each objective and the corresponding diameter values of the CCCs were found as 2.5 mm for circular CCC, 2.5 and 3.8 mm for the lower and greater diameters of the tapered CCCs, respectively. To that end, the printing process was completed using the most convenient printing parameters and the maximum error of the printing step was found less than 5% for both circular CCCs and tapered CCCs, which showed that the printing step was done with high-quality performance. Also, the maximum deviation between different strut diameters of the BCC lattices was measured at 0.06 mm, which was a highly satisfying value for the small diameters. 

Following the outputs of the current work, near-future research can focus on more complicated solutions such as (i) higher number of design variables, (ii) higher number of parameter sets, (iii) development of complex neural network structures for a better and faster performance mapping, (iv) integration of non-continuous optimization constraints during the thermal management of the injection mold, and (v) applying different types of lattice structures. All of the proposed future works will increase the solution procedures and design spaces of the CCC applications in plastic injection technology.

## Figures and Tables

**Table 1:** Properties of MS1 powder.

Powder Property	Result
Particle size	21–52 µm
Powder density	4.21 g/cm^3^
Solid density	8.1 g/cm^3^
Powder water content	18 ppm
Element (%weight)	Fe (Balance)-Ni (18.09)-Co (9.08)-Mo (4.86)-Ti (0.79)-Cu (0.02)-Cr (0.11)-Al (0.11)-Mn (0.04)-Si (0.08)-C (0.01)-P (<0.01)-S (<0.01)
Yield strength	1980 (MPa)
Tensile strength	2063 (MPa)
Elongation	4 (%)
Hardness	55 (HRC)

**Table 2:** The set of printing parameters in the DMLS process for the BCC-integrated tapered CCC.

Variable/Sample Code	A	B	C	D	E
Laser power (W)	$0.5\cdot P_{0}$	$P_{0}$	$2\cdot P_{0}$	$2\cdot P_{0}$	$2\cdot P_{0}$
Scan rate (mm/s)	$u_{0}$	$u_{0}$	$u_{0}$	$0.8\cdot u_{0}$	$0.67\cdot u_{0}$
Energy density factor	0.5	1	2	2.5	3

$P_{0}$ and $u_{0}$ are the parameters for printing SS 316L parts, which we prefer them as baseline parameters for printing of MS1, with the constant values of 60 W and 1594 mm/s, respectively.

**Table 3:** The target diameter values during the MAM process for CCCs.

Channel Type	D or D1 (mm)	D2 (mm)
Circular CCC	2.5	-
Tapered CCC	2.5	3.8
Tapered CCC with BCC lattice	2.5	3.8

**Table 4:** Printing quality check for CCCs according to the channel dimensions.

CCC Type	Locations/Diameters	1	2	3	4	5	6	7	Error (%)
Circular CCC	D_target_ (mm)	2.5							4.0
	D_real_ (mm)	2.42	2.46	2.40	2.42	2.44	2.42	2.46	
Tapered CCC	D_1-target_ (mm)	2.5							2.4
	D_1-real_ (mm)	2.44	2.48	2.44	2.44	-	-	-	
	D_2-target_ (mm)	3.8							2.6
	D_2-real_ (mm)	-	-	-	-	3.70	3.84	3.84	
Tapered CCC with BCC lattice	D_1-target_ (mm)	2.5							3.2
	D_1-real_ (mm)	2.42	2.46	2.44	2.48	-	-	-	
	D_2-target_ (mm)	3.8							1.6
	D_2-real_ (mm)	-	-	-	-	3.76	3.82	3.86	

**Table 5:** Printing quality check for BCC lattices according to the strut diameters.

	Diameters (mm)	d_1_	d_2_	d_3_	d_4_	Maximum Deviation
BCC 1	Target	0.50				0.06 mm
	Measured	0.44	0.50	0.52	0.44	
BCC 2	Target	0.50				0.06 mm
	Measured	0.42	0.52	0.46	0.44	
BCC 3	Target	0.50				0.06 mm
	Measured	0.48	0.46	0.50	0.44	

## Data Availability

Not applicable.

## Appendix A

**Table A1:** Computational simulation results for circular CCCs (Volume flow rate: L/min and Coolant temperature: Kelvin).

Volume Flow Rate/Coolant Temperature	1 (L/min)	3 (L/min)	5 (L/min)	7 (L/min)	10 (L/min)
	Cooling Time (s)/Temperature Non-Uniformity (K)/Pressure Drop (MPa)				
D = 2.1 mm					
288 (K)	5.6/5.82/0.02	5.4/6.62/0.14	5/6.93/0.39	4.5/6.93/0.75	3.9/7.01/1.49
290 (K)	5.7/5.49/0.02	5.5/6.52/0.14	5.1/6.45/0.39	4.6/6.54/0.75	4/6.62/1.49
293 (K)	5.9/4.99/0.02	5.7/5.7/0.14	5.3/5.88/0.39	4.7/5.96/0.75	4.2/6.03/1.49
296 (K)	6.1/4.49/0.02	5.9/5.14/0.14	5.4/5.31/0.39	4.8/5.39/0.75	4.3/5.45/1.49
298 (K)	6.3/4.41/0.02	6.1/4.77/0.14	5.6/4.93/0.39	4.9/5/0.75	4.5/5.07/1.49
D = 2.2 mm					
288 (K)	5.6/5.84/0.01	5.4/6.67/0.12	5/6.89/0.31	4.6/6.99/0.59	3.7/7.08/1.19
290 (K)	5.7/5.51/0.01	5.5/6.3/0.12	5.1/6.51/0.31	4.7/6.6/0.59	3.8/6.68/1.19
293 (K)	5.9/5.01/0.01	5.7/5.74/0.12	5.3/5.93/0.31	4.8/6.02/0.59	3.9/6.09/1.19
296 (K)	6.1/4.51/0.01	5.9/5.18/0.12	5.4/5.36/0.31	5/5.44/0.59	4/5.51/1.19
298 (K)	6.2/4.18/0.01	6/4.81/0.12	5.5/4.97/0.31	5.1/5.05/0.59	4.2/5.87/1.19
D = 2.3 mm					
288 (K)	5.5/5.87/0.01	5.3/6.72/0.09	4.9/7.19/0.25	4.4/7.05/0.48	3.6/7.14/0.96
290 (K)	5.6/5.53/0.01	5.4/6.34/0.09	5/6.56/0.25	4.5/6.66/0.48	3.7/6.74/0.96
293 (K)	5.8/5.03/0.01	5.6/5.78/0.09	5.2/5.98/0.25	4.6/6.07/0.48	3.8/6.15/0.69
296 (K)	6/4.53/0.01	5.8/5.22/0.09	5.3/5.4/0.25	4.8/5.48/0.48	3.9/5.55/0.96
298 (K)	6.1/4.2/0.01	6/4.85/0.09	5.4/5.01/0.25	4.9/5.09/0.48	4/5.16/0.96
D = 2.4 mm					
288 (K)	5.5/5.89/0.01	5.3/6.77/0.08	4.9/7.01/0.21	4.4/7.12/0.4	3.6/7.21/0.79
290 (K)	5.6/5.56/0.01	5.4/6.39/0.08	5/6.62/0.21	4.5/6.72/0.4	3.7/6.81/0.79
293 (K)	5.8/5.05/0.01	5.6/5.83/0.08	5.2/6.03/0.21	4.6/6.13/0.4	3.9/6.21/0.79
296 (K)	6/4.55/0.01	5.8/5.26/0.08	5.3/5.45/0.21	4.7/5.54/0.4	4/5.61/0.79
298 (K)	6.1/4.22/0.01	5.9/4.88/0.08	5.5/5.06/0.21	4.8/5.14/0.4	4.1/5.21/0.79
D = 2.5 mm					
288 (K)	5.4/5.93/0.01	5.2/6.93/0.06	4.8/7.08/0.17	4.3/7.19/0.31	3.6/7.29/0.61
290 (K)	5.5/5.59/0.01	5.3/6.45/0.06	4.9/6.68/0.17	4.4/6.82/0.31	3.7/6.8/0.61
293 (K)	5.7/5.09/0.01	5.5/5.88/0.06	5.1/6.09/0.17	4.5/6.31/0.31	3.9/6.2/0.61
296 (K)	5.9/4.58/0.01	5.6/5.31/0.06	5.2/5.59/0.17	4.7/5.63/0.31	4/5.8/0.61
298 (K)	6/4.05/0.01	5.8/4.72/0.06	5.4/5.25/0.17	4.9/5.37/0.31	4.1/5.5/0.61

**Table A2:** Computational simulation results for serpentine CCCs (Volume flow rate: L/min and Coolant temperature: Kelvin).

Volume Flow rate/Coolant Temperature	1 (L/min)	3 (L/min)	5 (L/min)	7 (L/min)	10 (L/min)
	Cooling Time (s)/Temperature Non-Uniformity (K)/Pressure Drop (MPa)				
Elliptical CCC					
288 (K)	5.3/6.35/0.05	5.1/7.52/0.39	4.9/7.55/1.06	4.5/7.46/2.04	2.9/6.8/4.11
290 (K)	5.4/5.83/0.05	5.2/7.1/0.39	5/7.12/1.06	4.7/7.02/2.04	3/6.38/4.11
292 (K)	5.5/5.3/0.05	5.4/6.68/0.39	5.1/6.69/1.06	4.8/6.58/2.04	3.1/5.96/4.11
294 (K)	5.6/4.78/0.05	5.6/6.26/0.39	5.3/6.26/1.06	4.9/6.14/2.04	3.2/5.54/4.11
296 (K)	5.8/4.27/0.05	5.7/5.83/0.39	5.4/5.82/1.06	5/5.71/2.04	3.3/5.13/4.11
298 (K)	6/3.77/0.05	5.9/5.41/0.39	5.5/5.39/1.06	5.1/5.27/2.04	3.5/4.73/4.11
Elongated CCC					
288 (K)	5.2/7.02/0.04	5.1/7.6/0.31	4.9/7.64/0.85	4.6/7.54/1.63	3.4/6.86/3.31
290 (K)	5.3/6.63/0.04	5.3/7.17/0.31	5/7.2/0.85	4.7/7.09/1.63	3.5/6.43/3.31
292 (K)	5.5/6.25/0.04	5.4/6.75/0.31	5.1/6.76/0.85	4.8/6.65/1.63	3.6/6.01/3.31
294 (K)	5.6/5.86/0.04	5.6/6.32/0.31	5.3/6.32/0.85	4.9/6.21/1.63	3.7/5.59/3.31
296 (K)	5.9/5.47/0.04	5.7/5.89/0.31	5.4/5.89/0.85	5/5.77/1.63	3.8/5.17/3.31
298 (K)	6.1/5.08/0.04	5.9/5.47/0.31	5.6/5.45/0.85	5.2/5.33/1.63	3.9/4.76/3.31
Triangular CCC					
288 (K)	5.5/6.9/0.06	5.2/7.44/0.48	5/7.47/1.29	4.6/7.37/2.51	3.2/6.72/5.02
290 (K)	5.8/6.52/0.06	5.3/7.02/0.48	5.2/7.03/1.29	4.7/6.93/2.51	3.3/6.3/5.02
292 (K)	5.9/6.14/0.06	5.5/6.61/0.48	5.3/6.61/1.29	4.8/6.5/2.51	3.4/5.89/5.02
294 (K)	6/5.76/0.06	5.6/6.19/0.48	5.4/6.18/1.29	4.9/6.07/2.51	3.5/5.47/5.02
296 (K)	6.1/5.38/0.06	5.8/5.77/0.48	5.5/5.76/1.29	5.1/5.64/2.51	3.6/5.07/5.02
298 (K)	6.3/4.99/0.06	5.9/5.35/0.48	5.6/5.33/1.29	5.3/5.16/2.51	3.7/4.67/5.02

**Table A3:** Computational simulation results for tapered CCCs (at the coolant temperature of 298 K and the volume flow rate of 10 L/min; units of D_2_ and D_1_ are mm) lattice.

D_2_ /D_1_	3 (mm)	3.25 (mm)	3.50 (mm)	3.75 (mm)	4.0 (mm)
	Cooling Time (s)/Temperature Non-Uniformity (K)/Pressure Drop (MPa)				
Tapered CCC					
2.1 (mm)	3.20/5.00/1.13	3.10/5.15/1.16	3.00/5.18/1.19	2.91/5.22/1.20	2.89/5.26/1.23
2.2 (mm)	3.20/4.99/0.95	3.10/5.16/0.95	3.00/5.19/0.96	2.91/5.22/1.01	2.89/5.23/1.05
2.3 (mm)	3.20/5.03/0.79	3.10/5.13/0.81	3.00/5.20/0.83	2.91/5.20/0.80	2.89/5.27/0.82
2.4 (mm)	3.20/5.01/0.65	3.10/5.13/0.66	3.00/5.19/0.68	2.91/5.20/0.67	2.89/5.27/0.69
2.5 (mm)	3.20/5.05/0.58	3.10/5.12/0.57	3.00/5.20/0.56	2.91/5.21/0.55	2.89/5.28/0.55
Tapered CCC with BCC Lattice					
2.1 (mm)	3.00/4.88/3.29	2.90/4.92/2.79	2.80/4.97/2.48	2.70/5.02/2.33	2.60/5.16/1.98
2.2 (mm)	3.00/4.89/2.59	2.90/4.92/2.38	2.80/4.98/2.11	2.70/5.02/1.98	2.60/5.14/1.75
2.3 (mm)	3.00/4.91/2.38	2.90/4.93/2.02	2.80/5.00/1.76	2.70/5.02/1.58	2.60/5.13/1.46
2.4 (mm)	3.00/4.88/2.03	2.90/4.95/1.74	2.80/5.04/1.58	2.70/5.07/1.37	2.60/5.10/1.27
2.5 (mm)	3.00/4.93/1.81	2.90/4.96/1.51	2.80/5.02/1.31	2.70/5.02/1.19	2.60/5.16/1.06
